# Efficacy of Cefiderocol against Carbapenem-Resistant Gram-Negative Bacilli in Immunocompetent-Rat Respiratory Tract Infection Models Recreating Human Plasma Pharmacokinetics

**DOI:** 10.1128/AAC.00700-17

**Published:** 2017-08-24

**Authors:** Shuhei Matsumoto, Christine M. Singley, Jennifer Hoover, Rio Nakamura, Roger Echols, Stephen Rittenhouse, Masakatsu Tsuji, Yoshinori Yamano

**Affiliations:** a Drug Discovery & Disease Research Laboratory, Shionogi & Co., Ltd., Toyonaka, Osaka, Japan; b Antibacterial Discovery Performance Unit, GlaxoSmithKline Pharmaceuticals, Upper Providence, Pennsylvania, USA; c Infectious Disease Drug Development Consulting, LLC, Easton, Connecticut, USA

**Keywords:** cefiderocol, S-649266, carbapenem resistant, rat respiratory tract infection model, recreating human PK

## Abstract

Cefiderocol (S-649266), a novel siderophore cephalosporin, shows potent activity against carbapenem-resistant Gram-negative bacilli. In this study, we evaluated the efficacy of cefiderocol against carbapenem-resistant Gram-negative bacilli (Pseudomonas aeruginosa, Acinetobacter baumannii, and Klebsiella pneumoniae) in immunocompetent-rat respiratory tract infection models recreating plasma pharmacokinetics (PK) profiles in healthy human subjects. A total of 6 clinical isolates (1 cephalosporin-susceptible P. aeruginosa isolate, 1 multidrug-resistant P. aeruginosa isolate, 2 multidrug-resistant A. baumannii isolates, and 2 carbapenem-resistant K. pneumoniae isolates) were evaluated. Four-day treatment with a human exposure of 1 g ceftazidime every 8 h as a 0.5-h infusion showed potent efficacy only against a ceftazidime-susceptible isolate, not against five ceftazidime-resistant isolates harboring carbapenemase. With cefiderocol, a human exposure of 2 g every 8 h as a 3-h infusion for 4 days produced a >3 log_10_ reduction in the number of viable cells of these carbapenem-resistant isolates in the lungs. When the infusion time was 1 h, bactericidal activity was also observed against all isolates tested, although for 2 of 5 carbapenem-resistant isolates, a 3 log_10_ reduction was not achieved. The difference in efficacy achieved by changing the infusion period from 1 h to 3 h was considered to be due to the higher percentage of the dosing interval during which free-drug concentrations were above the MIC (%*fT*_MIC_), as observed for β-lactam antibiotics. These results suggest the potential utility of cefiderocol for the treatment of lung infections caused by carbapenem-resistant P. aeruginosa, A. baumannii, and K. pneumoniae strains.

## INTRODUCTION

Multidrug-resistant (MDR) Gram-negative bacilli, such as MDR Pseudomonas aeruginosa (MDRP), MDR Acinetobacter spp. (MDRA), and carbapenem-resistant Enterobacteriaceae (CRE), represent a serious public health problem (1–3). P. aeruginosa and Acinetobacter baumannii are major pathogens causing severe infections in immunocompromised hosts, and about 13% and 63% of severe health care-associated infections caused by P. aeruginosa and A. baumannii, respectively, are reported to be caused by MDR strains (3). Infections caused by CRE have also been reported to cause high mortality, and the proportion of carbapenem-resistant strains among Enterobacteriaceae increased from 1.2% in 2001 to 4.2% in 2011 in the United States (4). There is a significant unmet medical need for the treatment of infections caused by these Gram-negative bacilli, because few treatment options are currently available. Colistin, an antibiotic developed in the 1950s, has recently regained a principal role in the treatment of infections caused by MDRP, MDRA, and CRE (5, 6). However, its use is limited due to its adverse-event profile and limited evidence of efficacy, and the emergence of colistin resistance has also been reported (7–10).

Cefiderocol (S-649266) is a novel siderophore cephalosporin discovered by Shionogi & Co., Ltd., that shows potent activity against Gram-negative bacilli, including MDRP, MDRA, and CRE (11, 12). The antibacterial activity of cefiderocol is due to its efficient outer membrane penetration through its utilization of the bacterial iron transport system (13) and its high stability in the presence of β-lactamases, including both serine- and metallo-type carbapenemases (14). In this study, we evaluated the efficacy of cefiderocol against MDRP, MDRA, and carbapenem-resistant Klebsiella pneumoniae (CRKP) in rat respiratory tract infection models recreating human plasma pharmacokinetics (PK).

## RESULTS

### Antimicrobial activity.

The cefiderocol MICs for the test strains were 0.125 to 8 μg/ml, while all test strains except for P. aeruginosa ATCC 27853 showed resistance to ceftazidime (CAZ) and meropenem (MEM), with MICs of >32 and ≥8 μg/ml, respectively (Table 1).

**TABLE 1:** MICs of cefiderocol, ceftazidime, and meropenem against test isolates

Isolate	Phenotype and/or carbapenemase^*a*^	MIC (μg/ml)		
		Cefiderocol	Ceftazidime	Meropenem
P. aeruginosa				
ATCC 27853	Ceftazidime susceptible	0.5	2	0.25
SR27001	MDR, IMP-1	2	>32	>32
A. baumannii				
1485176	MDR, OXA-51-like	0.125	>32	32
1485247	MDR, OXA-51-like	2	>32	8
K. pneumoniae				
VA-361	CR, KPC-2	4	>32	16
KI2	CR, NDM-1	8	>32	>32

a MDR, multidrug resistant; CR, carbapenem resistant.

### Determination of cefiderocol concentrations in the plasma of infected rats recreating human PK profiles.

**FIG 1:**
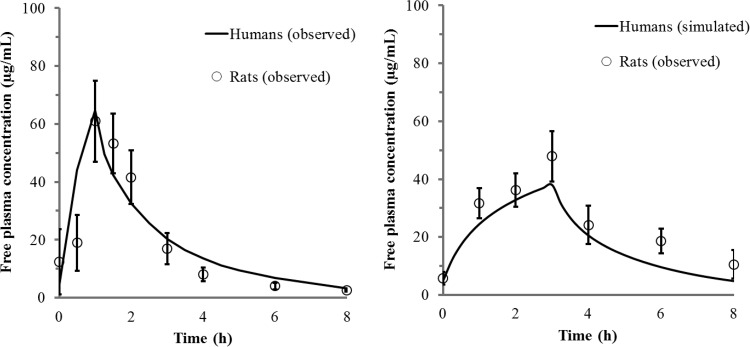
Free-drug concentration-time profiles in plasma for a 1-h infusion (left) or a simulated 3-h infusion (right) of 2 g cefiderocol in humans and rats. Each open circle represents the mean; error bars, standard deviations (*n* = 8). PK profiles in humans and rats were derived from healthy humans and infected rats (left) or from simulated humans and infected rats (right).

**FIG 2:**
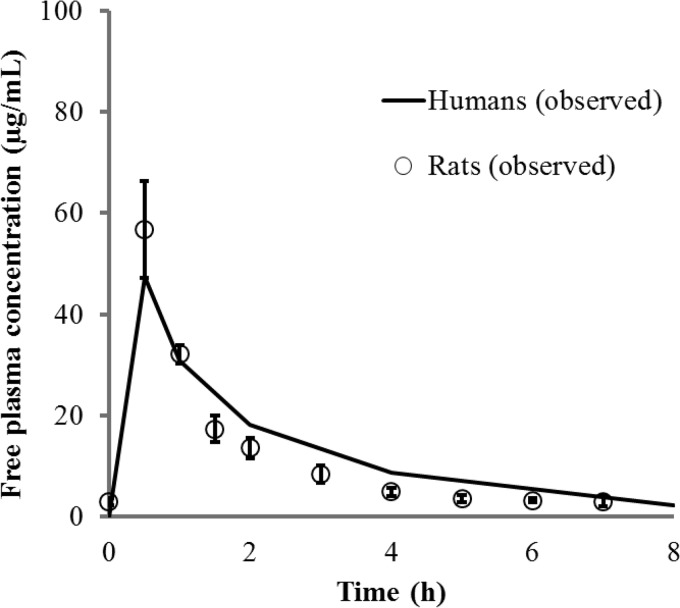
Free-drug concentration-time profiles in plasma for a 0.5-h infusion of 1 g ceftazidime in humans and rats. Each open circle represents the mean; error bars, standard deviations (*n* = 5).

The concentration-time profiles of free cefiderocol in the plasma of infected rats are shown in Fig. 1 and were compared with observed or simulated concentrations in human plasma following the administration of 2 g cefiderocol as a 1-h or 3-h infusion. The PK profiles of cefiderocol in human plasma were successfully recreated in the infected rats by use of a computer-controlled infusion system. The recreated human PK profiles of CAZ in the infected rats were also comparable to those in humans (Fig. 2).

### Efficacy of cefiderocol in rat respiratory tract infection models.

**FIG 3:**
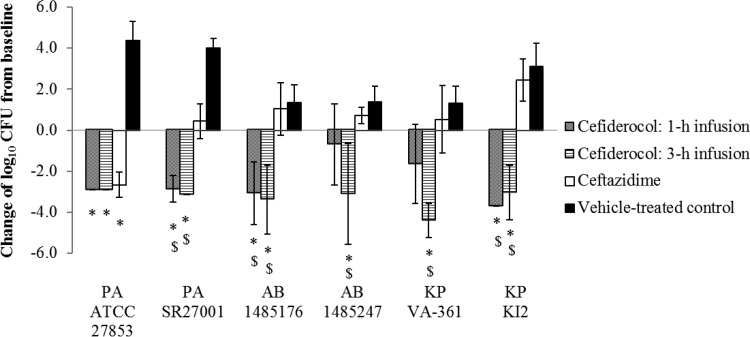
Efficacy of cefiderocol and ceftazidime against test isolates in rat respiratory tract infection models recreating human plasma PK. Data represent means ± standard deviations of results from 4 to 7 rats. The symbols below the bars indicate significant differences (*P* < 0.05) from the baseline control value (*) or from the result for ceftazidime ($) by Welch's *t* test. PA, Pseudomonas aeruginosa; AB, Acinetobacter baumannii; KP, Klebsiella pneumoniae.

All the data in the figures represent the means and standard deviations of results from 5 to 7 rats per group, except for K. pneumoniae isolate KI2, for which the 1-h baseline, 3-h cefiderocol infusion, and vehicle control results pertain to 3, 3, and 4 rats, respectively, and A. baumannii 1485176, for which the vehicle control result pertains to 4 rats. Across all studies, 6% of rats (11 of 181) had to be excluded due to technical issues. No outliers were identified and no data points removed retrospectively; thus, 170 data points were included in the efficacy results. The mean bacterial counts at the initiation of therapy were (4.2 ± 0.5) log_10_ CFU/lung for P. aeruginosa and (5.6 ± 0.7) log_10_ CFU/lung for A. baumannii and K. pneumoniae (3 or 5 rats were used per isolate). The changes in the log_10_ CFU counts per lung after 96 h from those at the initiation of therapy are shown in Fig. 3. In the rats recreating the human PK for 2 g cefiderocol given every 8 h (q8h) as a 1-h infusion, reductions of 0.7 log_10_ CFU to 3.7 log_10_ CFU in the counts of 5 carbapenem-resistant isolates (including P. aeruginosa, A. baumannii, and K. pneumoniae) were observed. In contrast, in the rats recreating the simulated prolonged-infusion regimen as a 3-h infusion, potent efficacy—with reductions ranging from 3.04 log_10_ CFU to 4.41 log_10_ CFU—was observed against all 5 carbapenem-resistant isolates tested. Human exposure to 1 g CAZ q8h as a 0.5-h infusion for 96 h showed potent efficacy only against the cephalosporin-susceptible P. aeruginosa isolate ATCC 27853. The extrapolated percentages of the dosing interval during which free-drug concentrations were above the MIC (%*fT*_MIC_) achieved with a human exposure of 2 g cefiderocol q8h given as 1- and 3-h infusions to infected rats are shown in Table 2.

**TABLE 2:** Extrapolated %*f T*_MIC_^*a*^ achieved with a human exposure of 2 g cefiderocol every 8 h as a 1- or 3-h infusion in infected rats

Cefiderocol MIC (μg/ml)	%*f T*_MIC_ with an infusion time of:	
	1 h	3 h
0.125	100	100
0.25	100	100
0.5	100	100
1	100	100
2	100	100
4	75	100
8	50	100

a %*fT*_MIC_, percentage of the dosing interval during which free drug concentrations are above the MIC.

## DISCUSSION

Cefiderocol, a novel siderophore cephalosporin, shows potent *in vitro* activity against Gram-negative bacilli, including MDRP, MDRA, and CRE (11, 12). In this study, we evaluated the efficacy of cefiderocol against MDRP, MDRA, and CRKP in rat respiratory tract infection models recreating human plasma PK.

The *in vitro* activity of cefiderocol against 9,205 clinical isolates, including 1,530 P. aeruginosa, 1,148 A. baumannii, 428 Stenotrophomonas maltophilia, and 6,087 Enterobacteriaceae isolates collected in 2014 to 2015 in North America and Europe, showed that MIC_90_ values of cefiderocol against P. aeruginosa, A. baumannii, and Enterobacteriaceae were 0.5, 1, and 0.5 μg/ml, respectively (15). Remarkably, 99.6% of all the isolates showed MICs of ≤4 μg/ml. These results indicate that the test isolates used in the present study were the most challenging isolates for cefiderocol, with MICs higher than the MIC_90_. The bactericidal activity against all test isolates in rat respiratory tract infection models recreating human plasma PK profiles strongly suggested that cefiderocol may be a promising candidate for treating patients with pneumonia caused by carbapenem-resistant Gram-negative bacteria, such as P. aeruginosa, A. baumannii, and K. pneumoniae.

The efficacy of cefiderocol against some test isolates was enhanced when the human PK were recreated by administering cefiderocol as a 3-h infusion rather than a 1-h infusion. Such enhanced efficacy was observed for A. baumannii 1485247 and K. pneumoniae VA-361, with MICs of 2 and 4 μg/ml, respectively. Like that of other β-lactam antibiotics, the efficacy of cefiderocol is well correlated with %*fT*_MIC_ (16). It is well known that administration via prolonged infusion may lead to increased efficacy for β-lactam antibiotics by increasing the %*fT*_MIC_ values (17). In addition, it was calculated that the %*fT*_MIC_ values against the strain with a MIC of 4 μg/ml were 75% and 100% when cefiderocol was administered by 1-h and 3-h infusions, respectively. These results suggested that the important PK/pharmacodynamic (PD) parameter for predicting the efficacy of cefiderocol would be %*fT*_MIC_, as with other β-lactam antibiotics.

Although neutropenic murine thigh infection models are frequently used for evaluating the efficacy of antibiotics, rat respiratory tract infection models have also been used for evaluating the efficacy of antibiotics in terms of human PK profiles (18–23). However, only the rat allows for semipermanent cannulation, which provides access for controlled human PK drug exposure. There are some differences between murine thigh infection models and rat respiratory infection models. First, the immunosuppression levels are different: the mice are immunocompromised, while the rats are immunocompetent. Second, the treatment durations are different: 1-day treatment in murine models versus 4-day treatment in rat models. In order to establish the infection in immunocompetent rats, bacteria were enveloped in molten agar to prevent killing by phagocytic cells. With these procedures, lower challenge doses (4 log_10_ CFU to 5 log_10_ CFU/lung) of the test strains led to the establishment of respiratory tract infections that were characterized by >1-log_10_ CFU increases within 24 h. Four days of treatment were used to evaluate the bacterial eradication that would result from longer treatment. Since CAZ showed efficacy only against a CAZ-susceptible strain in the recreated human PK profile (Fig. 3), we believe that this rat model is appropriate for the evaluation of the efficacy of antibiotics in human PK.

In summary, we evaluated the *in vivo* efficacy of cefiderocol against MDRP, MDRA, and CRKP in rat respiratory tract infection models recreating human plasma PK. A human exposure of 2 g cefiderocol q8h showed potent efficacy against the challenging clinical isolates with MICs higher than the MIC_90_. These results suggest the potential utility of cefiderocol for the treatment of lung infections caused by carbapenem-resistant P. aeruginosa, A. baumannii, and K. pneumoniae strains.

## MATERIALS AND METHODS

### Antimicrobial compounds.

Cefiderocol and cefiderocol-*d_12_*, synthesized by Shionogi & Co., Ltd. (Osaka, Japan), were used. Ceftazidime (CAZ) and meropenem (MEM) obtained from USP (Rockville, MD) were used for MIC determinations. In *in vivo* efficacy studies, the marketed human intravenous formulation of CAZ (Fortaz) was used as a reference cephalosporin to show that our animal model is appropriate for evaluation of the efficacy of antibiotics in terms of human PK.

### Test organisms.

During the initial *in vitro* screening of cefiderocol, 60 clinical isolates of MDRP and 45 clinical isolates of MDRA were studied. From these global clinical isolates, 5 MDR isolates (1 MDRP isolate from Japan, 2 MDRA isolates from the United States, and 2 CRKP isolates from the United States and Europe) and 1 cephalosporin-susceptible isolate (P. aeruginosa ATCC 27853), with cefiderocol MICs ranging from 0.125 to 8 μg/ml, were selected for *in vivo* efficacy studies. MDR was defined as resistance to ceftazidime, imipenem, and ciprofloxacin (24). MDRP, MDRA, and CRKP isolates harbored various genes encoding carbapenemases: the IMP-1, OXA-51-like, and KPC-2 (25) or NDM-1 genes.

### MIC determination.

According to the recommendations of the Clinical and Laboratory Standards Institute (CLSI) (26), ceftazidime and meropenem MICs were determined by a broth microdilution method. Cefiderocol MICs were determined using iron-depleted cation-adjusted Mueller-Hinton broth (ID-CAMHB). This methodology was approved by the CLSI meeting in January 2016. ID-CAMHB was prepared as follows: 100 g of Chelex 100 resin (Bio-Rad, Hercules, CA) was added to 1,000 ml of autoclaved CAMHB, and the mixture was then incubated at room temperature for 2 h. The incubated medium was filtered with a 0.22-μm filter, and the pH was adjusted to 7.2 to 7.4, followed by supplementation with 20 to 25 μg/ml of Ca^2+^, 10 to 12.5 μg/ml of Mg^2+^, and 0.5 to 1 μg/ml of Zn^2+^. MIC studies were conducted with three replicates, and the modal MICs are shown in Table 1.

### Animals.

Pathogen-free male Sprague-Dawley rats (Charles River, Raleigh, NC) weighing approximately 200 g were used. Animals were allowed access to food and water *ad libitum*. All procedures met the standards of the American Association for the Accreditation of Laboratory Animal Care (27).

### Preparation of cannula-implanted rats.

Three to 6 days prior to the initiation of dosing, the inferior jugular vein (for dosing) and/or the carotid artery (for sampling blood) was cannulated under isoflurane (4%) anesthesia. Cannulae exited the rats through a dorsal incision and were encased in a metal spring tether. The tether provided protection for the cannulae and served as a swivel system to allow free movement of the rat around the cage. All cannulated rats received subcutaneous flunixin meglumine (Banamine) at a dose of 1.1 mg/kg of body weight at 24 and 48 h postsurgery for pain relief.

### Establishment of respiratory tract infection.

Respiratory tract infections were established according to previous reports on the evaluation of antibiotics using an immunocompetent-rat infection model recreating human plasma PK (18–23). To prepare the inocula for infection, thawed stock cultures were grown overnight in brain heart infusion (BHI) broth (Becton, Dickinson, and Company, Sparks, MD) and were mixed with molten nutrient agar (BBL) at a ratio of 1:9 at 41°C immediately prior to the initiation of the infection procedure. Respiratory tract infection was induced by first anesthetizing immunocompetent rats with a combined intramuscular injection of ketamine hydrochloride (40 mg/kg) and xylazine (5 mg/kg) and then injecting 0.1 ml (P. aeruginosa) or 0.2 ml (A. baumannii or K. pneumoniae) of the inoculum into their lungs via nonsurgical intratracheal intubation (28).

### Recreating human PK profiles in infected rats.

The PK profile observed for healthy subjects on day 10 after dosing with 2 g cefiderocol every 8 h (q8h) as a 1-h infusion (29, 30) was recreated in the infected rats. The mean values for the maximum concentration of the drug in plasma, the concentration in plasma at time zero (trough), and the terminal half-life in humans were 154 μg/ml, 11.4 μg/ml, and 2.77 h, respectively. The human PK profile for prolonged-infusion cefiderocol (3-h infusion) was simulated from the PK parameters for a 1-h infusion by using two-compartment model analysis. GW-BASIC was used to create a simple personal-computer (PC) interface with the infusion pumps. This was a custom-designed program that controlled the pumps by resetting infusion rates every 15 min. The infusion rates required to recreate the exposure profiles were established in preliminary PK studies and were manually entered into the GW-BASIC program prior to the initiation of the efficacy studies. For CAZ, the recreated human PK profile was based on the plasma concentration-time curve observed with a single 1-g dose given as a 0.5-h infusion (according to the CAZ package insert). The PK profiles of cefiderocol and CAZ were corrected based on the protein binding ratios in humans and rats; thus, the recreated profiles represent free-drug concentrations in plasma for both species.

In order to compensate for species differences (between rats and humans) in elimination kinetics, the elimination half-life in rats was modified by using an intravenous infusion. After subcutaneous administration of a single bolus at 50 mg/kg to infected rats, the area under the concentration-time curve (for plasma) was 67.2 μg · h/ml, and the terminal half-life was 0.636 h. A computer regulated the infusion rates of the dosing solution at regular intervals over 8 h in order to recreate the concentrations in the plasma of healthy human subjects. The flow rates were reset q8h to simulate a three-times-daily dose. Dosing solutions of cefiderocol and CAZ (20 and/or 40 mg/ml in 0.9% saline) were freshly prepared each day.

### Determination of drug concentrations in the plasma of infected rats recreating human PK profiles.

According to the established flow rate program, the dosing solution was infused into 8 (for cefiderocol) or 5 (for CAZ) rats infected with P. aeruginosa ATCC 27853. On the second day of dosing, blood was collected at various times after the start of the infusion. Whole blood (approximately 150 μl) was collected from each rat via the carotid artery cannula into EDTA-coated or heparinized tubes. Blood was centrifuged for 2 min at 14,000 rpm to separate plasma. A 50-μl aliquot of each plasma sample was diluted with 50 μl of 200 mM ammonium acetate (pH 5.0). The diluted samples (100 μl) were frozen immediately on dry ice and were stored at −80°C prior to analysis. Concentrations of the drug in plasma were determined by the validated liquid chromatography-tandem mass spectrometry (LC–MS-MS) method. Samples were deproteinated with the cefiderocol-*d_12_* internal standard as a working solution in 0.2% trifluoroacetic acid (TFA) in methanol. Double blanks were composed of 0.2% TFA in methanol spiked into acidified rat plasma. For plasma calibration, appropriate concentrations of cefiderocol were spiked into rat plasma to give eight standards from 0.25 to 250 μg/ml. Quality control (QC) samples were prepared by spiking rat plasma with cefiderocol to achieve final concentrations of 0.3 (low QC), 10 (medium QC), and 200 (high QC) μg/ml. The LC–MS-MS system comprised an Agilent 1290 HPLC system in tandem with an Agilent 6430 triple-quadrupole MS in electrospray ionization mode. Chromatographic separation was performed using a Halo C_18_ column (inside diameter, 2.1 mm; length, 30 mm; particle size, 2.7 μm; Advanced Materials Technology) with a gradient using mobile phases of 10 mM ammonium formate (pH 3.0) and acetonitrile–isopropanol–0.1% formic acid (40:40:20) at a flow rate of 0.5 ml/min. Cefiderocol concentrations were obtained using LC–MS-MS monitoring the product ion transitions of *m/z* 752 and *m/z* 285.1. The analysis run time was 3.2 min. The assay was linear over a range from 0.25 to 250 μg/ml (*r*^2^, >0.999). The interday coefficients of variation for cefiderocol QC samples ranged from 2.1 to 5.5%, and mean accuracy was 99.7% ± 7.0% (range, 82.9 to 116.3%). The lower limit of quantification was 0.25 μg/ml.

### Efficacy of cefiderocol in rat respiratory tract infection models.

Treatment was started at 1 h postinfection for all organisms except P. aeruginosa, as per standard protocol. Due to the severity of P. aeruginosa infection, a lower inoculum was used so as to reduce mortality; treatment was started 2 h postinfection to allow CFU counts to reach a higher baseline level. Treatments were continued for 96 h for the recreated human PK profile (6 animals/dosing group). Vehicle control animals received saline at a constant flow rate of 0.4 ml/h for the duration of the study (6 animals/dosing group except for K. pneumoniae KI2, for which the vehicle control group contained 7 rats). Lungs from all animals were harvested 96 h after the initiation of therapy. Any rat that did not complete 96 h of treatment due to technical issues (i.e., complications from anesthesia, stalled infusion pumps, or blocked cannulae) was eliminated from the analysis. To determine the bacterial counts in the lungs (expressed as log_10_ CFU per lung), rats were euthanized by a carbon dioxide overdose, and the lungs were excised aseptically and were homogenized in 1 ml 0.9% saline. Viable bacterial cells were counted on Trypticase soy agar plates containing 5% sheep blood after overnight incubation at 37°C. The lower limit of detection was ≤1.2 log_10_ CFU/lung. In addition to the above-mentioned treatment and vehicle control animals, 6 untreated rats were assigned to the untreated control group; they were harvested at the initiation of dosing and served as baseline controls.

### Determination of protein binding.

The protein binding values of cefiderocol in humans and rats were determined by the ultrafiltration method. Briefly, cefiderocol dissolved in phosphate-buffered saline (PBS) (pH 7.4) was added to freshly collected human or rat plasma to prepare a 10-μg/ml test sample. The test sample was incubated at 37°C in a water bath for 15 min and was dispensed into ultrafiltration devices (Centrifree YM-30; Millipore, Bedford, MA). After centrifugation for 15 min at 37°C and 1,800 × *g*, the filtrate was collected. The protein binding ratio was calculated from the cefiderocol concentrations in the test sample before centrifugation and in the filtrate after centrifugation. The protein binding values in humans and rats were 58% and 47%, respectively. The protein binding values of CAZ in humans and rats (21% and 20%, respectively) were taken from the literature (31).

### Data analysis.

The percentage of the time from 0 to 8 h during which free-drug concentrations remained above a stipulated MIC value (%*fT*_MIC_) was extrapolated using the free-drug concentration-time profiles in the plasma of infected rats. The change in bacterial density was calculated as the change in the log_10_ CFU count/lung obtained for the treatment group and the vehicle control group at 96 h postinfection from that observed for the baseline control. Statistical analysis of differences between the treatment groups and the baseline control groups was performed using Welch's *t* test. Additionally, statistical analysis of differences between the cefiderocol treatment groups and the CAZ treatment group was performed using Welch's *t* test. The *P* value for significance was <0.05.
